# Beyond the tracked line of sight - Evaluation of the peripheral usable field of view in a simulator setting

**DOI:** 10.16910/jemr.12.3.9

**Published:** 2021-04-26

**Authors:** Jan Bickerdt, Hannes Wendland, David Geisler, Jan Sonnenberg, Enkelejda Kasneci

**Affiliations:** Volkswagen AG, Wolfsburg, Germany; Audi AG, Ingolstadt, Germany; Eberhard-Karls-Universität, Tübingen, Germany

**Keywords:** Eye tracking, gaze, attention, saccades, fixations, visual perception, simulation, automotive

## Abstract

Combining advanced gaze tracking systems with the latest vehicle environment sensors opens up new fields of applications for driver assistance. Gaze tracking enables researchers to determine the location of a fixation, and under consideration of the visual saliency of the scene, to predict visual perception of objects. The perceptual limits, for stimulus identification, found in literature have mostly been determined in laboratory conditions using isolated stimuli, with a fixed gaze point, on a single screen with limited coverage of the field of view. The found limits are usually reported as hard limits. Such commonly used limits are therefore not applicable to settings with a wide field of view, natural viewing behavior and multi-stimuli. As handling of sudden, potentially critical driving maneuvers heavily relies on peripheral vision, the peripheral limits for feature perception need to be included in the determined perceptual limits. To analyze the human visual perception of different, simultaneously occurring, object changes (shape, color, movement) we conducted a study with 50 participants, in a driving simulator and we propose a novel way to determine perceptual limits, which is more applicable to driving scenarios.

## Introduction

In a highly dynamic context, such as maneuvering a vehicle, multiple challenges arise. Due to the complexity of the driving environment, a large number of constantly changing objects must be observed and analyzed simultaneously in order to infer driver’s perception of these objects (1, 2, 3). As perception limits are usually determined under laboratory conditions, on a single screen, with limited coverage of the participants field of view the found, hard perception limits cannot fully cover the peripheral detection of sudden events found in scenarios with a broad field of view and natural viewing behavior. To find a more suitable way to determine and represent perception probabilities for the whole Field of View (FOV), we conducted a driving simulator study with 50 participants’ using a 149° projection, a multi-camera, and a wide field of view gaze tracking system (4).

In addition to technical challenges, multiple new regulations mandate the integration of driver monitoring systems into Advanced Driver Assistance Systems (ADAS) in future production cars (5, 6, 7). More specifically, future cars must be able to recognize driver states like distraction, inattention and driver availability in order to adapt ADAS’ warnings and active assistance (8, 9, 10).

We argue that future ADAS will be able to estimate which surrounding objects are unperceivable from the drivers’ perspective only by knowing drivers’ perception limits. Other use cases for in-vehicle visual attention detection include, but are not limited to, driver perception specific warnings and attention guiding.

To develop Advanced Driver Assistance Systems (ADAS) challenging scenarios need to be identified and tested. The first step to test the developed system is usually a completely simulated scene. If the system performs as expected in a simulation, it is crucial to incrementally make the system tests more realistic and determine the systems reaction to a real driver, and real world, as well as the drivers’ reaction to the assistance provided by the system.

One way to implement the second step of testing is system integration into a real car and testing in a controlled, simplified environment with dummy obstacles. This setup is the best way to safely test the systems reaction to the real world and the measurement inaccuracies, sensor errors and real-world influences like weather, lighting etc. Testing complete, complex scenarios this way is very costly, and challenging and therefore is rarely done during the early steps of the development process.

Another option for system testing is the use of driving simulators. These allow for complete control over the scenes complexity and parameters like weather and lighting while allowing for exact repeatability of the relevant scenarios and the possibility to quickly implement and deploy changes to the scene and the system. Additionally, a driving simulator guarantees safety for the test subject and the used material. Therefore this has become one of the most common methods for rapid, early, user centered prototyping.

**Figure 1:**
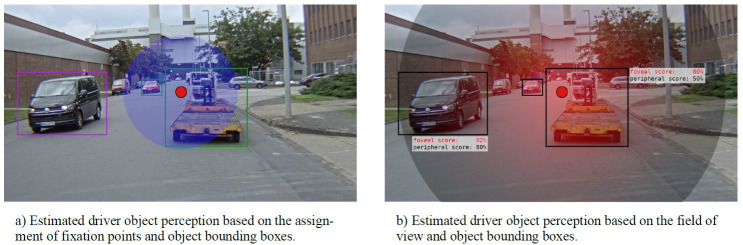
A typical driving situation with a fused eye tracking system and traffic object recognition. The red dot indicates the estimated fixation point in the scene. Bounding boxes indicate detected traffic objects. The left figure (a) shows a direct matching of fixation points to detected vehicles. Objects whose bounding box does not contain a fixation point are evaluated as not perceived. The figure on the right side (b) sketches the usage of the entire field of view to determine whether an object was perceived or not. Resulting in a two different scores for the perception probability of an object, one based on its proximity to the fixation point and the other one based on its visual appearance and the driver’s capability to perceive said appearance.

When analyzing the drivers’ observation behavior, eye tracking can be used. A large part of object perception is peripheral and can therefore not be modeled by conventional, fixation-based methods (see figure 1). To use algorithms for perception estimation including outer peripheral vision, it is essential to know the perception probabilities in a setup with a wide field of vision and multiple simultaneously occurrence stimuli, such as a driving simulator.

## Related Work

Several notable works have already studied the field of view for different visual features. More specifically, the found perception boundaries have been determined by the conclusion of user studies under laboratory conditions (11, 12, 13, 14) and modeling the eye (4, 12, 15, 16, 17, 18, 19). Something most of the aforementioned studies have in common is the use of only a few objects at the same time or the use of distractions which are clearly distinguishable from the relevant features. Furthermore, usually only one visual parameter is varied at any given moment. For the given setup this is insufficient as in a driving scenario multiple simultaneously occurring and changing stimuli are present. One common approach to determine an objects perception probability is calculating its visual saliency with visual models inspired by the aforementioned studies (20, 21, 22). Multiscale feature maps are, in these models, used to extract rapid changes in color, intensity and orientation. On videos, motion is also considered. The found features are combined and an individual score for each pixel or object can be calculated (23).

A widespread approach in eye tracking is the estimate of the user’s visual axis, by use of a calibrated system and mapping the detected pupil to a scene. Whether an object has been examined in the scene is often determined by an assignment of fixation points using fixed boundaries around an object or region of interest (24, 25).

However, the estimation of such a visual axis is not fully representative but rather a simplification as the human visual perception is by far not limited to a straight line of sight. In fact, the human eye opens up to ~135° vertical and ~160° (binocular ~200°) horizontal of perception (4). Objects in this visual field reflect or emit light which encounters the eye, is focused onto the retina by a lens and gets absorbed by photoreceptors. Depending on the retinal location, different capabilities of perception are available. Located along the visual axis is the fovea. Within this area lie the majority of photoreceptors for both chromatic and contrast perception, resulting in an improved ability to perceive these features. This is a comparatively small area (≤1.5mm Ø vs. 32mm Ø of the complete retina (26, 27), but provides the most detailed perception, and typically corresponds to the center of our visual attention under daylight conditions. With increasing eccentricity to the visual axis, the ability of chromatic perception decreases and achromatic photoreceptors are dominating the perception. These are more sensitive and react faster than chromatic photoreceptors, but form larger and more overlapping fields, resulting in lower resolution and contrast in the peripheral vision (4, 12, 15, 16, 17, 19). Therefore, depending on the excerpt of the visual field, different kinds of visual features are extracted, emphasized, and transmitted to the brain via the optic nerve. Higher cognitive processes then evaluate and filter the extracted scene content based on its semantic relevance (28, 29). Whether and how an object is actually perceived and presented to consciousness strongly depends on the characteristics of the emitted visual stimuli from the scene, the capabilities of the affected area on the retina, and their relevance in the currently performed task or intention.

**Figure 2:**
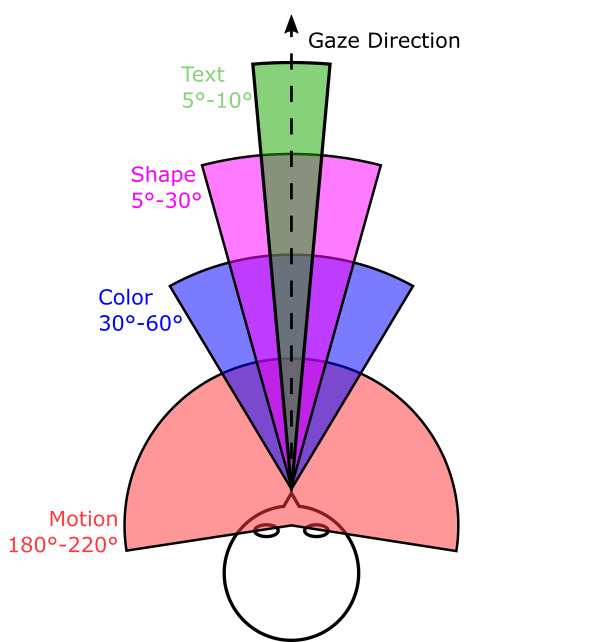
Schematic representation of the horizontal field of view. Movements are almost perceptible over the entire field of vision, while colors are mostly perceived, and can only be identified in the inner ±30°. Detailed perceptions such as shapes or texts, which depend on a very high resolution and sharp contrasts, are therefore only available in the middle of the field of view. (4, 13, 11, 14)

This leads to different angles of perception for various perceptible characteristics. Figure 2 shows that e.g. movement can almost be identified in the complete field of view. The perceptible faculty of color contrast differs with increasing eccentricity. While in the foveal area mainly red-green contrast dominates, the maximum perception of blue-yellow contrast is in the parafoveal area.

By determining the visual area in which information can be identified without head or eye movement, also known as the Useful Field of View (UFOV) (30, 31), participants’ perception is compared. To determine the UFOV-Score, usually three or more different tasks are used (32). These tasks consist of six basic events with small variations for each task: ready, position, stimulus/stimuli, noise, response, and feedback. First the participant is asked to declare a state of readiness by pushing a button. Second, if necessary, the possible stimuli positions are shown. Depending on the setup, roughly one second later the stimuli are shown for 16.67-500 ms. The length of the stimuli visibility is meant to be long enough to allow for the participants to become aware of its existence, but too short to allow for its fixation. To eliminate any visual “after effect”, in the next step some source of visual noise is shown at all possible stimuli positions. This is especially important for the center task which relies on object identification. Afterwards, the participant is required to give a response specific for the executed task. Lastly, feedback about the input is given, when appropriate.

In detail, most tasks are as follows:

- Focused Attention / Processing Speed: Central Task. For this task an object, for example a random letter is shown in the monitor center. Because only one position is available the position event can be eliminated. Goal of this task is object identification. A response on the identification correctness is given. The duration of the object visibility is gradually decreased until identification is impossible for the participant. This task is used to determine the participants’ processing speed.
- Focused Attention: Peripheral Task. For this task multiple stimuli positions are possible and are shown on the monitor throughout the whole task. Identification of the stimulus position is the goal of this task. No feedback is given.
- Divided Attention. This task is a combination of the previous tasks. A center stimulus and a peripheral stimulus appear at the same time. Participants are supposed to identify the center stimulus and the peripheral stimulus' position. Feedback is given for the center stimulus.
- Selective Attention. This task is similar to the last task, but in this configuration a number of distractions are visible alongside the relevant stimuli.

These are very openly defined with regards to stimuli and distractions, which means they could be adapted to determine a specific UFOV for perception parameters like motion, form and color by choosing the right kind of stimuli and distraction.

Nevertheless, some limitations exist for our use case. The use of a specific central fixation point would lead to static viewing behavior which is unnatural compared to the viewing behavior in real driving tasks. The use of eye tracking would allow for the estimation of the fixation point at any time and enable a more natural viewing experience. Also the use of noise and clearly defined, manually started time frames for stimuli appearance make testing a lot of different stimuli positions very time consuming. Therefore, we choose to implement our own approach.

It should also be noted, that the traditional UFOV describes the limits for stimuli identification. Peripheral vision, being able to notice a stimuli without being able to identify it, extends over a broader region of our FOV. Furthermore it is important to know, that traditional UFOV tests focused attention on a limited set of object positions in a narrow part of the FOV (33). Additionally, the UFOV is often specified as homogenous with clear cutoffs.

Danno et al. examine the influence a driving task can have on the peripheral vision. They implement a real-time UFOV algorithm (rUFOV), and test the accuracy of the determined edges of the UFOV with a driving task in a simulator (34). This approach is much more realistic than the traditional UFOV but comes with drawbacks. Even though Danno et al. examine the influence which different risk levels have on the participants rUFOV, risk perception and the resulting stress levels vary individually (35). Therefore, using a driving task could lead to incomparable changes in the participants perception.

To our knowledge the peripheral Useful Field of View (pUFOV), the complete FOV in which stimuli can be perceived, has never been tested in a setup, with a wide FOV, multi-stimuli and a natural viewing behavior.

## Experimental Setup

**Figure 3:**
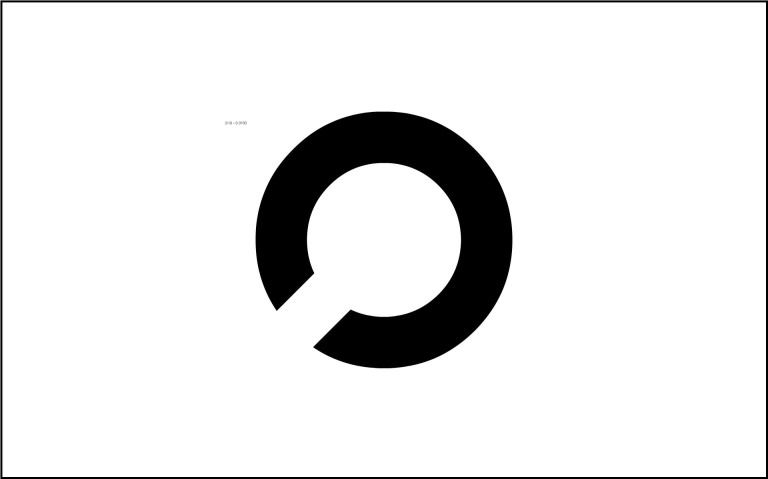
Landol C used by FrACT

To quantify the effect of multiple simultaneously occurring stimuli on visual perception in a driving simulator setting, we conducted a user study with 50 subjects. The used simulator comes with the advantage of being designed to cover a huge part of the driver’s field of view. This enables a maximum of evaluable peripheral vision while at the same time allowing complete design freedom for the visual scene (not only for driving scenes). The simulator was equipped with three 3.05m x 1.89m screens (see figure 3). This corresponds to 147° x 39° coverage of the drivers’ field of view. Each of the screens is fitted with a color calibrated "Barco F12 WUXGA VizSim" projector (36). The projectors have a resolution of 1920px x 1200px, which results to an overall minimal spatial resolution of 91.8 arcsec and 117 arcsec respectively. Under ideal conditions, the human eye has a maximum resolution of 2 arcsec (37). Therefore, even with suboptimal eyesight, every participant would be able to perceive everything shown on the screens. As the presence of visual obstructions, in a driving simulator, might have an effect on the participants’ visual perception, (38) a car with a built-in and calibrated SmartEye Pro remote eye tracking system was placed in the center of the driving simulator. Consisting of four NIR Basler GigE cameras with 1.3 MP resolution, the eye tracking system achieves an accuracy of up to 0.5 degrees in estimating the gaze direction (39, 40) under optimal conditions. A remote system was used because it does not restrict the subject in its head movement, which leads to a more natural viewing behavior.

In order to ensure the best possible data quality, participants with visual aids were not invited to participate. To ensure that participants with visual impairment did not influence the outcome of the study, a series of pre-tests has been conducted to determine the overall visual performance. Color perception was tested with 17 Isihara color test plates (41). Two further tests assessed the participants' eyesight and contrast perception.

**Figure 4:**
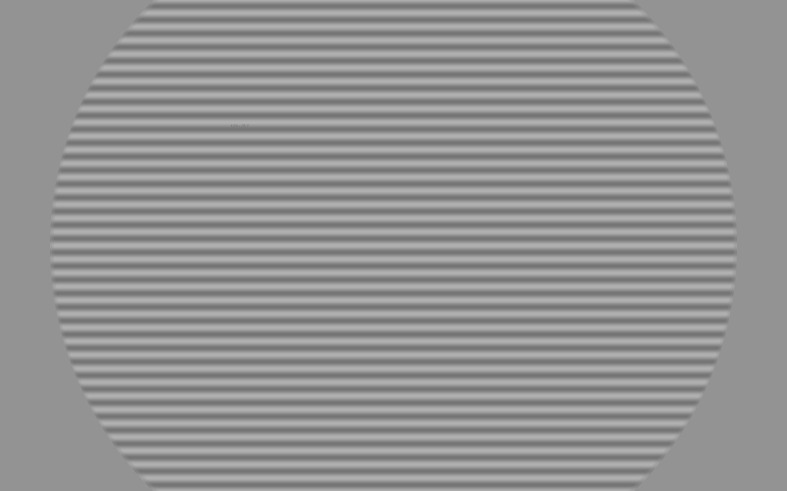
Contrast grating used by FrACT

All tests were performed at a distance of 257cm in front of a color and contrast calibrated "Eizo ColorEdge CG245W" monitor (38). The tests were run using the software "Freiburger Visual Acuity Test" (FrACT) by Michael Bach (42). It has been shown that FrACT provides reproducible results comparable to the results of the Bailey-Love Chart and the regular Landolt C Charts (43). The visual acuity was measured using Landolt C optotypes (44) as implemented in FrACT (see figure 4). Participants had to find the gap in 18 consecutively presented, increasingly smaller Landolt C optotypes. The minimal displayable opening was one pixel, which translates to a minimum spatial resolution of 0.36 arcmin.

**Figure 5:**
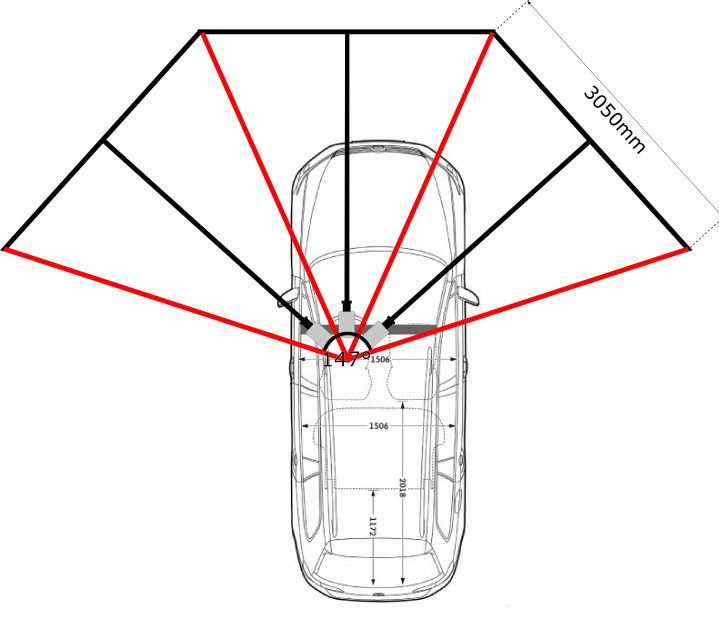
The three screens are aligned at an angle of 151° to each other. The distance to the driver in the center is about 3,75m. Each screen has a size of 3.05m x 1.89m, which leads in a coverage of the field of view of 147° x 39° (compare figure 7).

The participants' perception of contrast was assessed using a grid of horizontal, vertical or diagonal lines (see figure 5). The shown grid occupied 10° of the participants field of view, with a spatial frequency of 5 cpd and a minimal Michelson contrast of 51% . The contrast between these lines was decreased until the minimum perceptible contrast was found.

**Figure 6:**
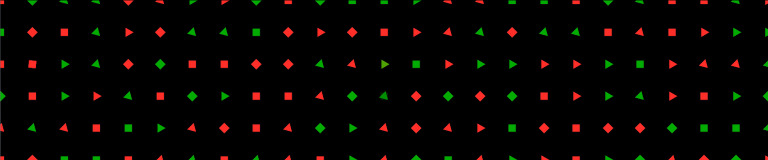
Example of a used feature grid. The displayed features may change in color and shape or move as depicted in Figures 8-10. Different feature changes may occur simultaneously, but never changes of the same type.

**Figure 7:**
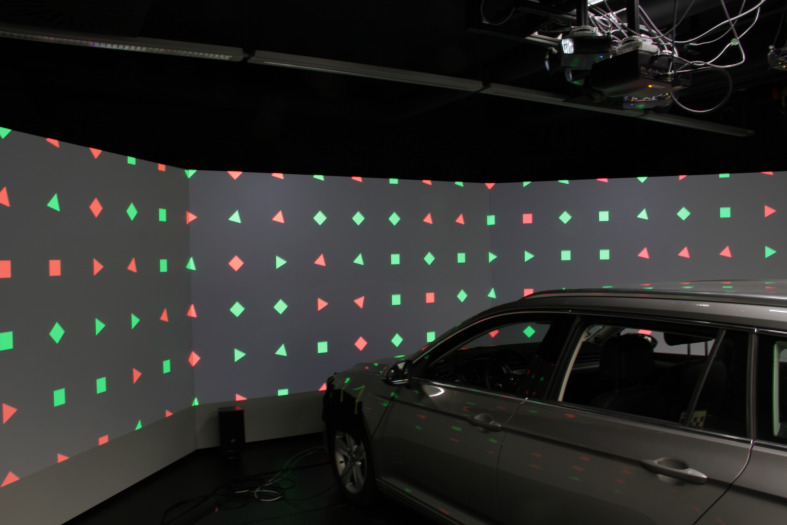
Simulator setup

The main task of the study consisted of three consecutive videos (each of them had a duration of 5 minutes) with 150 synthetic objects of size 40px x 40px each, arranged in a 25 x 6 matrix (see figure 6). The object size resulted in a spatial resolution of 480-720 arcmin, depending on the objects’ position. Decreasing towards the sides of each canvas and increasing towards the center. No measures have been taken to account for the cortical magnification effect, because with a natural, free viewing behavior, object size would need to be adjusted on the fly, which could lead to unwanted visual effects. The objects have been randomly generated with regards to shape (square / triangle), color (green / red), and orientation (rotated by 0° or 45°) and evenly distributed over the screens with a distance of 200 px (see figures 6 & 7). This arrangement is based on findings in "Feature Integration Theory" by Treisman et al. (45)to prevent visual grouping by color, shape, or position. Furthermore, the used colors green and red are defined in the LAB color space (46), which allows a transition between those colors while preventing a change in brightness.

**Figure 8:**
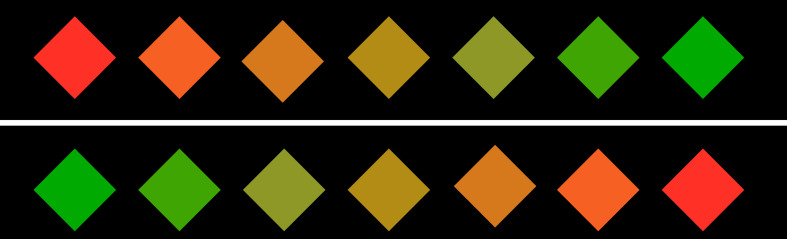
color change sequence

**Figure 9:**
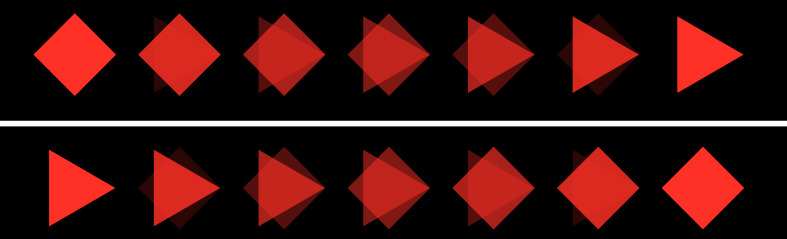
form change sequence

**Figure 10:**
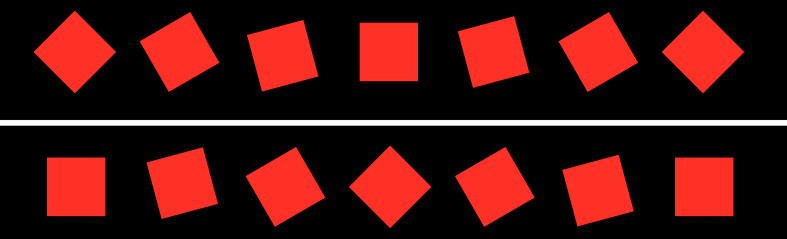
motion sequence

In order to determine the sensitive areas of the visual field for movement, shape and color changes, three different feature changes were implemented:

- The sensitivity to color changes was tested by a transition from red to green or vice versa. The color manipulation was carried out only in one of the color channels of the lab color space, which ensures a constant luminosity. At the same time, it allows color manipulation in one of the perceptible complimentary colors. (see figure 8)
- The perception of shapes was examined by a transition from triangles to squares and vice versa. (see figure 9)
- The sensitivity to movements was examined by wiggling of the features around 45° (see figure 10).

**Figure 11;:**
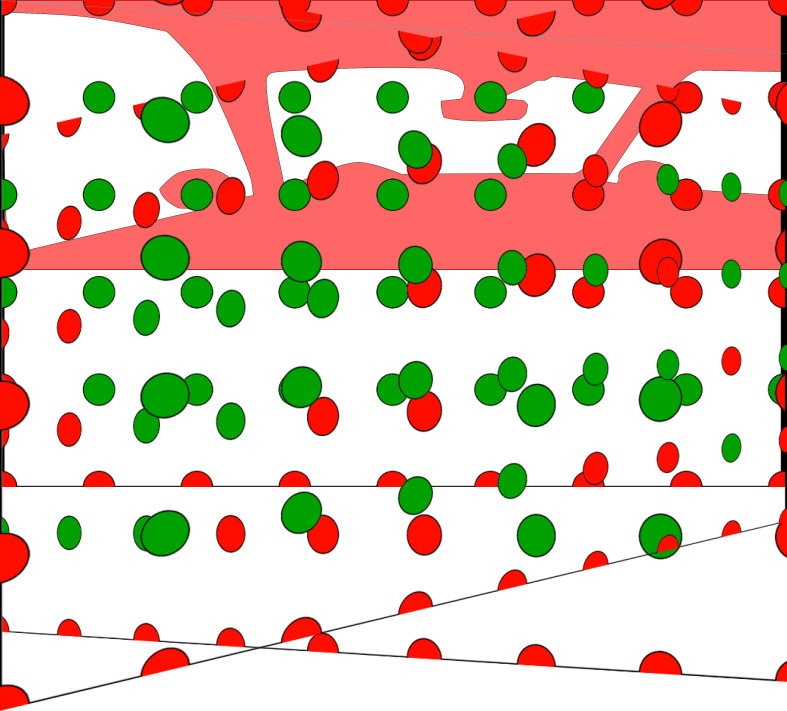
This figure shows boxplots for the mean measurement error caused by the eye tracking setup. It is divided into screenby- screen error and overall error. Included in brackets are the number of datasets which produced usable data in the calibration test vs. the total number of datasets.

These features and feature changes have been chosen because they have been shown to have a big impact on the saliency of objects and are perceivable in different areas of the FOV (4, 12, 15, 16, 17, 19, 20, 21).

**Figure 12:**
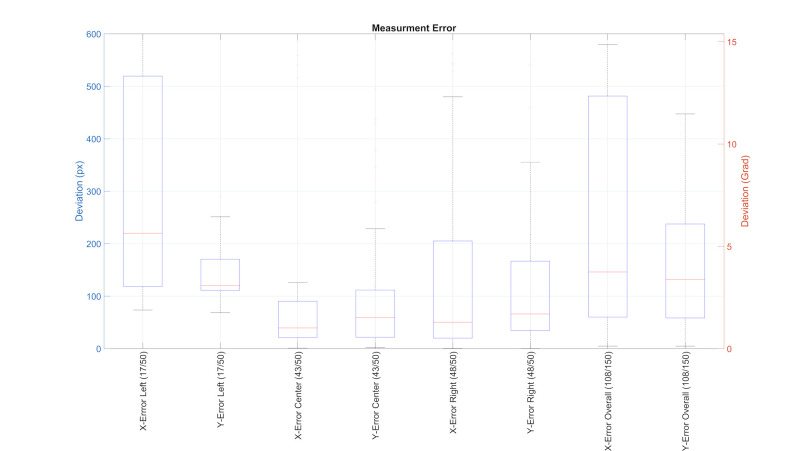
The red overlay indicates the occlusions by the vehicle chassis. Reference points and feature changes were only placed on green positions, to be well visible from the driver’s positions.

In order to prevent training and habituation effects as well as randomly created biases, a total of three videos were created and permuted in their order. The three types of feature changes were present in each of the videos. Taking into account the occlusion caused by the vehicle (see figure 12), the feature changes were applied randomly to the visible, displayed objects. Each change was visible between 1 and 2 seconds. The same types of change were never active at the same time, but with a maximum pause of 1 second to each other. During the duration of a video, a total of 140 events of each change type were visible.

**Figure 13:**
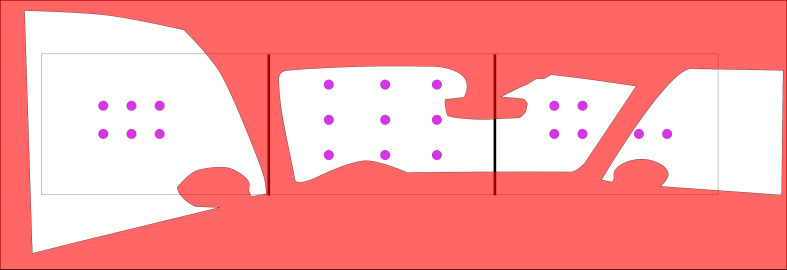
Calibration points

To ensure a maximum quality of the gaze tracking signal, the system was individually gaze calibrated for each participant using 21 reference points (see figure 13). Before the actual experiment videos started, the participants were instructed to gaze straight ahead into the center of the middle screen. If a feature change was noticed, the corresponding object should be looked at and acknowledged by pressing a button. The gaze should then be directed back to the center of the middle screen. To allow for a more natural viewing behavior, no central fixation point was used. After this instruction, the possible types of changes, as well as the arrangement of the features, were demonstrated by means of a training video. Afterwards, participants were instructed about the feature changes to look out for by the study supervisor and the first video was started. A two minute break between the videos was used to give the participants some time to rest, and to announce the next feature change to look for. The order of the relevant feature change, like the order of the videos, was randomized to suppress possible effects of a particular sequence. The experiment ended, when a participant completed all three videos. At the end of the study, the participants received a gift for their involvement.

## Evaluation

Perception rate, speed, and angle are the metrics used to compare visual feature perception in literature (4, 31, 34). Therefore, the focus of the data evaluation was on the detection rate of object changes, the reaction speed, as well as the saccade amplitude when a feature change was detected. The detection rate was determined by counting the number of correct acknowledged object changes. Two reaction times were measured. The time between start of the change event and the first look at it, and the confirmation by pressing the key. In other words, the time required by the participant to determine that the feature change under consideration was consistent with the search task. The distance to the visual axis, at the time of the first perception, was calculated by the saccade amplitude towards the corresponding feature, regarding a time window from the beginning of the feature change until the confirming keystroke. The accuracy of the calibrated eye tracking system was calculated by the mean deviation of the 21 reference points to the reported gaze points. This lead to an average error of ±3.9° (±150px) on the horizontal and 3.4° (±130px) on the vertical axis, across all participants. Figure 11 shows that the highest accuracy is achieved on the center screen and then descends to the right and left. This is due to the horizontal arrangement of the four cameras of the Eye Tracking system (one to the left of the driver, three to the right of the driver). If the participant was looking at the screen in the middle, the participants’ eyes were usually visible in all four cameras. However, if the head was pointed towards the right screen, the face was usually only visible in 3 cameras, and on the left screen it was usually only visible on 2 or less cameras.

Due to the constant noise caused by the eye tracking system setup, a direct assignment of fixation points to the presented features was not reasonable in most cases and would lead to a jittering gaze signal. In most cases, the noise arising from the construction of the eye tracking system setup did not allow a direct assignment of fixation points to the presented features. In order to determine a useful saccade amplitude, it was assumed that the displayed features were the only possible fixation points (the background was darkened and offered no contrast). The gaze signal was then aligned to the features using a Markov model combined with a random walk. Each of the features on the canvas was defined as a state of the Markov model. The resulting state vector v defines the relative and unnormalized likelihood that the currently measured fixation belongs to the corresponding feature. The transition probability from one state to another is defined by the difference in distance between the measured fixation point and the feature position displayed on the canvas:

(1) $$T_{f_{i}{\text{,}f}_{j}}=\left(1-e^{-\frac{\left|g_{t}-f_{j}\right|}{\sigma_{T}}}\right)-\left(1-e^{-\frac{\left|g_{t}-f_{i}\right|}{\sigma_{T}}}\right),$$

Where $g_{t}$ is the fixation point at the time t and $T_{f_{i}{\text{,}f}_{j}}$ is the transition likelihood from the feature $f_{i}$ to $f_{j}$, or respective the likelihood of a saccade from feature $f_{i}$ to $f_{j}$. With each additional measured fixation point the transition matrix is recreated and one iteration of the random walk is performed:

(2) $$v_{t}=T\times \;v_{t-1},$$

Where $v_{t}$ is the Markov state at iteration t. The aligned fixation point ${g\prime }_{t}$ at time t is then extracted as the feature with the maximal likelihood in $v_{t}$. Whenever g′ changed, the period between ${g\prime }_{t-1}$ and ${g\prime }_{t}$ was counted as a saccade (a string of saccades in a similar direction was counted as a single saccade).

All but one participant made less than four mistakes in the Ishihara test. For this study, this was considered as sufficient color perception. Since the data of this participant showed no negative impact on the overall performance, the data was kept in the data set for further analysis. The contrast test was always performed with the minimal perceived Michelson contrast of 0.51% and all participants scored better than 0.01 logMAR in the eyesight test.

The ratio of female to male participants was 42:58 with an average age of 35.5 years(s = 7.7).

## Results

**Figure 14:**
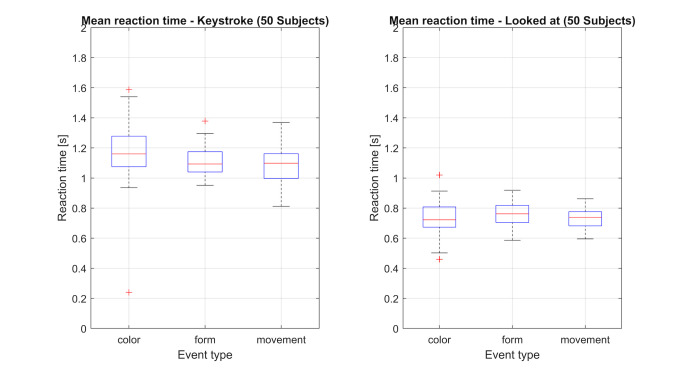
Boxplot of the measured reaction times for the identification of the three different object changes. The plot on the left visualizes the time needed between start of an object change and confirmation of its perception by button press. The plot on the right shows the time required to look at a changing object.

Two essential findings can be derived from the evaluation:

First, the mean time needed until the key is pressed for Color vs. Shape (C-S), and Color vs. Movement (C-M) and the mean time needed until the object is looked at for Shape vs. Movement (S-M), is significantly different (see Table 1). Second, as shown in figure 14 there is only a slight difference, of roughly 0.05-0.1 seconds, in the reaction time between the individual object modification types.

**Figure 15:**
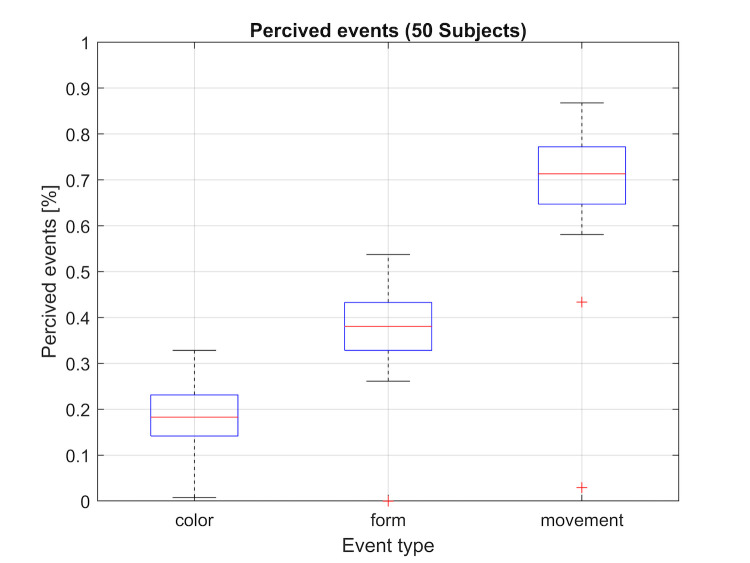
Length of the maximum saccade amplitude towards the feature corresponding to the object change in pixel and degree respectively.

**Table 1:** Results of the ANOVA calculated to examine H0: “There is no significant difference in the reaction time between the different types of changes.” (P≤0.05 is considered significant)

	Mean	
	Keypress	Gaze
C-S	P<0.05	P=0.06
S-M	P=0.3	P<0.05
C-M	P<0.01	P=0.6
C-S-M	P=0.06	P=0.08

C = Color; S = Shape; M = Movement

**Table 2:** Results of the ANOVA calculated to examine H0: “There is no significant difference in ratio of perceived changes between the different types of changes.” (P≥0.05 is considered significant)

	Perceived Changed
C-S	P<0.001
S-M	P<0.001
C-M	P<0.001
C-S-M	P<0.001

C = Color; S = Shape; M = Movement

With a reaction time of 0.7-0.8 seconds and a change duration of 1-2 seconds, differences in the recognition rate cannot be attributed to missed changes due to a too long reaction time.

However, the recognition rates and most of the measured mean and maximum saccade amplitudes differ significantly between the tested features:

**Table 3:** Results of the ANOVA calculated to examine H0: “There is no significant difference in the maximum/mean visual angle over which the different types of changes are perceived.” (P≥0.05 is considered significant)

	Mean		Maximum	
	Canvas	Angle	Canvas	Angle
C-S	P=0.35	P=0.41	P<0.001	P<0.001
S-M	P<0.001	P<0.001	P<0.001	P<0.001
C-M	P<0.001	P<0.01	P<0.001	P<0.001
C-S-M	P<0.001	P<0.001	P<0.001	P<0.001

C = Color; S = Shape; M = Movement

**Figure 16:**
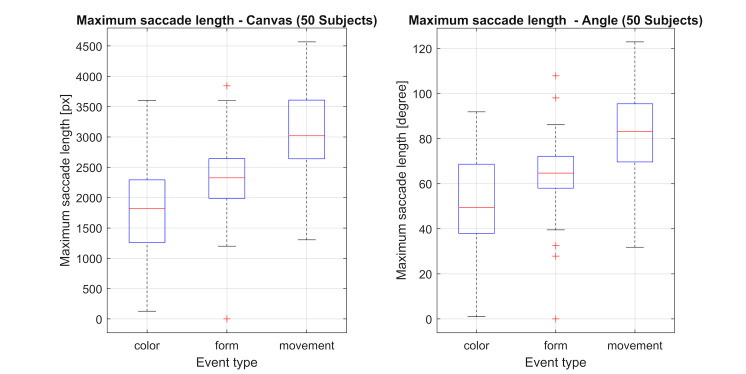
Recognition rate of the three features

Figure 16 and table 2 shows significant differences in the number of perceived feature changes between the three different feature types. An average number of 18%, 38% and 71% of the characteristic changes for color change, shape change and movement were observed.

Looking at figures 14 & 15 and table 3, some of these differences can be explained. Even though the mean saccade amplitude for color and form changes are not significantly different, there are significant differences in the maximum saccade amplitude for the different feature changes.

**Figure 17:**
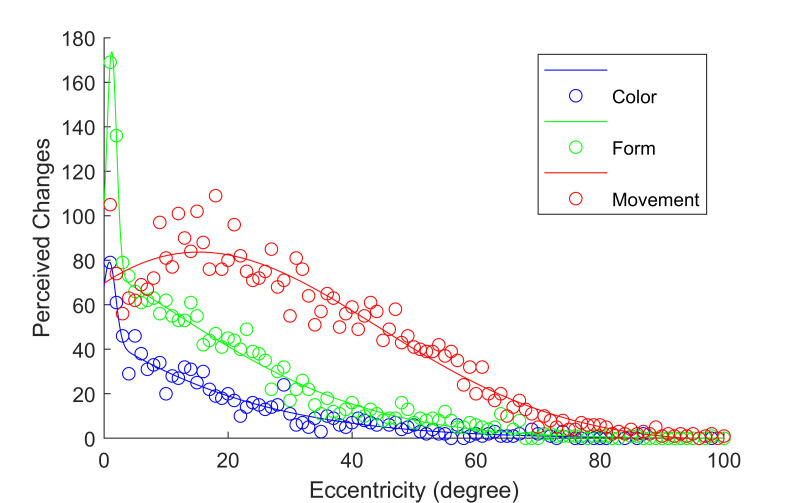
Perceived changes of the three features as Gaussian graphs, fitted to the perceived object changes eccentricity

A more intuitive visualization of the differences is given by figure 17. Even though color and form changes are visible over a similar section of the FOV, the recognition rate for form changes is higher over the whole FOV. Movement is detectable over a broader part of the FOV and the detection rate is higher in the parafoveal and peripheral area. Only in the foveal area, the detection rates for color and form surpass the detection rate for movement. A possible explanation of this effect is based on the structure of the visual system. As the resolution in the center of the foveal region increases, the number of connected parvocellular cells (p-cells) increases and the number of connected magnocellular cells (m-cells) decreases. Because m-cells react faster and stringer to movement, the perception of movement is slightly reduced compared to its maximum.

## Discussion

On the first glance, figure 1 and the determined pUFOV are contradictory, because the limits of the UFOV are a lot smaller than the limits of the pUFOV. This has two reasons. Firstly, the limits of the UFOV are determined by stimulus identification and the limits of the pUFOV are determined by stimulus detection. Secondly the stimuli used for the pUFOV are different. Therefore the resulting limits are not directly comparable.

With a measured average mean aperture angle of 164°, the perception of motion is higher than the 149° covered by the simulator. Since the feature changes were randomly distributed over the available objects and the participant's gaze was mostly directed to the center of the center screen, the average, maximum feature change distance would be about 74.5°, which would corresponds to a pUFOV of 149°. Considering the average measurement error of 3.5° (figure 13), the measured pUFOV could be considered the maximum measurable pUFOV for the used setup.

As figure 17 shows, average max and mean saccade amplitudes only provide limited information about the perception probability. A Gaussian based representation of the found probabilities should be used.

There are two ways to further verify the findings. Firstly a study similar to the lab based studies could be conducted with color changes, form changes and movement similar to the features proposed in this paper to determine whether the found perception probabilities still apply. Secondly, after focusing on more realistic features for this publication, another study could be conducted in a simulator setup similar to the one used for this paper. This time the tasks should exactly match the ones commonly used in literature (form recognition, color recognition, form recognition) to determine the perception probabilities for these stimuli.

The next interesting steps to further verify the validity of this papers’ findings would be to test the found perception limits with recorded real world scenes or driving simulation scenes. It would be wise to remove the driving task for the first iteration of this study to avoid effects which are caused by the driving task itself.

## Conclusion

In this work, we conducted a study to quantify the driver's perception of three types of visual features, color, shape, and movement across a wide field of vision with multiple simultaneous visual stimuli. 50 participants were asked to watch three 5-minute videos, each showing a series of randomly generated features in a 25x6 grid. During the process, three different types of possible feature changes occur. The task was to identify and mark all feature changes of one of the three types. The recorded gaze data was mapped to the corresponding objects using a Markov model. The saccade amplitude calculated with this method was used as an indicator at which angle from the focal point the feature change was perceived in the peripheral field of view. In addition, reaction times and perception rates for the type of changes were calculated.

The results show, that it is not sufficient to use a homogenous UFOV to predict the perception probability of an object is not enough. Different object characteristics have individual FOV with different perception probabilities for different eccentricities. Small changes in the way an object is presented can significantly change how likely it can be perceived. We recommend using a Gaussian representation of the perception probability in dependence of the stimulus eccentricity.

### Ethics and Conflict of Interest

The author(s) declare(s) that the contents of the article have been reviewed by an internal ethics review process and that there is no conflict of interest regarding the publication of this paper.
